# The influence of ergodicity on risk affinity of timed and non-timed respondents

**DOI:** 10.1038/s41598-022-07613-6

**Published:** 2022-03-08

**Authors:** Arne Vanhoyweghen, Brecht Verbeken, Cathy Macharis, Vincent Ginis

**Affiliations:** 1 Data Lab, Vrije Universiteit Brussel, 1050 Brussels, Belgium; 2 Business technology and Operations, Vrije Universiteit Brussel, 1050 Brussels, Belgium; 3 Mobility, Logistics, and Automotive Technology Research Centre, Vrije Universiteit Brussel, 1050 Brussels, Belgium; 4 School of Engineering and Applied Sciences, Harvard University, Massachusetts, 02138 USA

**Keywords:** Human behaviour, Applied mathematics, Statistical physics

## Abstract

Expected values are the metric most often used to judge human decision-making; when humans make decisions that do not optimize expected values, these decisions are considered irrational. However, while convenient, expected values do not necessarily describe the evolution of an individual after making a series of decisions. This dichotomy lies at the core of ergodicity breaking, where the expected value (ensemble average) differs from the temporal average of one individual. In this paper, we explore whether the intuition behind human decision-making optimizes for expected values or instead takes time growth rates into account. We do this using several stated choice experiments, where participants choose between two stochastic bets and try to optimize their capital. To evaluate the intuitive choice, we compare two groups, with and without perceived time pressure. We find a significant difference between the responses of the timed and the control group, depending on the dynamic of the choices. In an additive dynamic, where ergodicity is not broken, we observe no effect of time pressure on the decisions. In the non-ergodic, multiplicative setting, we find a significant difference between the two groups. The group that chooses under time pressure is more likely to make the choice that optimizes the experiment’s growth rate. The results of this experiment contradict the idea that people are irrational decision-makers when they do not optimize their expected value. The intuitive decisions deviate more from the expected value optimum in the non-ergodic part of our experiment and lead to more optimal decisions.

## Introduction

Are people rational decision-makers? When economic thinkers first studied this question, they conceptualized the “homo economicus,” a rational agent making rational decisions. However, the field soon discovered a major flaw with the concept homo economicus, as it did not accurately depict real-life human behavior. Consequently, a first tweak to the model was added under the guise of a new variable: humans want to optimize the utility of the outcome, a subjective but consistent measure in many decision-making experiments^1^. Unfortunately, the use of utility to model real-life decision-makers can lead to paradoxes, e.g., Allais paradox^2^ and inconsistencies, e.g., with the Von Neumann–Morgenstern axioms^3,4^. These shortcomings led to the development of alternative models, the most prevalent of which is prospect theory^5^. Today, both utility theory and prospect theory are widely used to model human decision-making. Intriguingly, at their core, both approaches use expected values to model human decisions and, based on that metric, both conclude that humans are irrational, albeit (mostly) internally consistent decision-makers. The question arises whether expected values guide us to the correct decision in the first place.

When studying dynamical processes, a convenient and often made assumption is the one of ergodicity. This assumption, rooted in statistical physics, states that the long-time average of an entity in a system approaches the ensemble average (expected value) of a system. This assumption allows us to take time out of consideration and estimate the long-time average using the ensemble average. However, is this assumption warranted for the economic processes we observe? That is precisely the question that Ole Peters puts forth in the 2019 paper: ‘the ergodicity problem in economics’^6^. This seemingly straightforward question offers many exciting venues for research, e.g., the origins of inequality^7,8^, or the usefulness of GDP/capita^9^. The variety of scientific questions are bundled under the umbrella of “ergodicity economics”. Our contribution focusses on the central question put forth by Peters^6^. To what extent do economic actors base their decisions on non-ergodic processes rather than ergodic ones? The main point of interest here is the discrepancy between expected values and the long-time averages for individuals and its impact on judgments of rationality. An illustrative example of a process that leads to this type of dissonance is the multiplicative wealth dynamic. Suppose, e.g., that you can increase your capital by 50% or decrease it by 40%, in a bet where both outcomes have an equal likelihood. Should you take the bet? What if you get to play a 1000 times?

Both classical behavioral economics^10^ and ergodicity economics^6,11^ would tell you that you should not take the bet. However, the reasoning as to why is remarkably different in both theories. Classical behavioral economics^10^, which focuses the brunt of its arguments on the expected value of the bet (here 5% gain^6,12^), concludes that you are probably not interested in the bet because of irrational biases and heuristics. Both expected utility theory^13^ and prospect theory^5^ tell us that economic agents rarely consider the monetary outcomes of the bet as it is but rather use the monetary outcomes as inputs for an idiosyncratic utility/value function. If this utility/value function outweighs losses over gains (i.e. loss aversion)^14–16^ the prospect of winning 50% is outshined by the dread of losing 40%. Both prospect theory and expected utility theory tell us that heuristics, biases, and bounded rationality^17^ bog down intuitive judgments, which leads us astray from the most optimal path.

**Figure 1:**
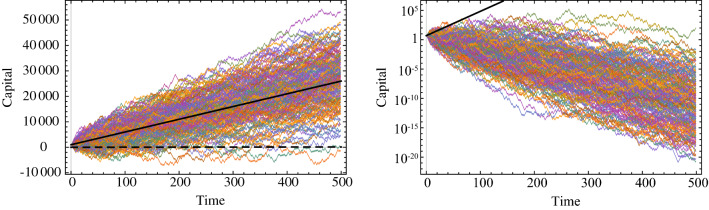
The left panel illustrates the capital of 200 individuals partaking in an additive scenario for 500 repetitions. The right panel illustrates the capital of 200 individuals participating in the multiplicative scenario for 500 repetitions. In both figures, the black line depicts the expected outcome of the bet. While the expected value provides a reasonable proxy for the evolution of the individuals’ capital in the ergodic (additive) setting, it is clear that this is not the case in the non-ergodic (multiplicative) setting.

In contrast, ergodicity economics tells us that in a non-ergodic setting, the expected value should be of little interest to the individual. This because it represents what would happen to the system for n $\rightarrow$ $+\infty$ individuals playing the bet once, rather than reflecting the more relevant information for the decision, namely what would happen to the wealth of one individual when t $\rightarrow$ $+\infty$. The latter attribute is represented by the time-average growth rate, here: $\ln {(1.5 \times 0.6)^\frac{1}{2}}\approx -5$%, for which $\ln$ represents the ergodic transformation^6^. Ergodicity economics concludes that you will not be interested in the bet because of very rational reasons, realizing that the bet will lead to personal ruin in the long term. We illustrate this process in Fig. 1. Notice that, unlike classical behavioral economics, the decision not to take the bet has nothing to do with irrational biases and heuristics in ergodicity economics, but rather its focus lies on the mathematical properties of the wealth dynamic in the given situation.

The question now remains, *which paradigm reflects real-world decision-makers? Do individuals care about time averages or expected values*?

A first and so far single attempt to answer this question was by Meder et al.^18^. In their experiment, a group of respondents (n = 18) had to repeatedly choose between 2 opposing bets (600 choices) in two different settings: in the additive dynamic (linear, ergodic process), the outcome of the bets was added to a respondent’s capital, whereas in the multiplicative scenario (exponential, non-ergodic process), the outcomes of the bets were multiplied with the respondent’s capital. To provoke intuitive decision-making in these settings, Meder et al.^18^ put respondents under time pressure and represented bet outcomes with images that had no direct association to the numeric values. Their findings can be summarised as follows: A respondent’s relative risk aversion parameter $\eta$ (Isoelastic Utility) does not remain constant but shifts in agreement with the experienced bet dynamic. This shift indicates that people use time averages, rather than ensemble averages, as a heuristic for decision making, which is in line with the predictions from ergodicity economics^6^. Despite these exciting findings, the experiment of Meder and colleagues^18^ has been criticized for the use of the stationary isoelastic utility function in a dynamic setting^19^. Doctor and colleagues point out that methods such as dynamic programming^20,21^ would have been more appropriate. Intrigued by the results from Meder et al.^18^, we set out to further explore the question of which paradigm reflects real-world decision-makers?

The dual setting, comparing additive vs. multiplicative scenarios, proposed by Meder et al.^18^ and Peters^6^, form the backbone of our stated preference experiment. This dual setting juxtaposes the focal points of both ergodicity economics and classical behavioral economics. Indeed, classical behavioral economics predicts a constant degree of risk aversion regardless of the dynamics of wealth accumulation. In contrast, ergodicity economics predicts a shift in decision-making preferences in line with the long-time average when ergodicity is broken. Another analogy between our experiment and the one from Meder et al.^18^, is that respondents had to identify their preferred bet among given bet couples. In our work, we introduced three additional dimensions to the experiment.

Firstly, we introduce a control group to verify the effect of time pressure on responses. We believe this is a relevant inclusion, as there is no consensus regarding the impact of time pressure on risk attitudes. Young and colleagues^22^ as well as Busemeyer and colleagues^23^ propose that time pressure increases a respondent’s propensity for risk, which is especially so for bets with which a respondent can expect to gain. Conversely, Zur and Breznitz^24^ found that respondents under time pressure became wearier of the adverse outcomes of a bet.

Secondly, we do not assume a specific utility function. Instead, we use our set-up to distinguish risk taking from risk averse decisions and as such focus our analysis on the frequency of taking the safe bet over the risky bet. To unambiguously distinguish between risky and safe bets, we designed the bet couples in such a way that both bets had equal expected values but different variance. Incidentally, this set-up allows for easy differentiation between optimal and non-optimal behavior according to time-average growth. The bets had the same expected value and time-average growth in the additive setting, which means none of the bet should dominate. However, bets with lower variance and equal expected value -the safer bets- always yielded a higher time-average growth than their more risky counterparts in the multiplicative setting.

Thirdly, we employ a description based setup (showing the actual numbers of the bet) in contrast to the experience based setup (where the values of the bet are learned over time by exploration) employed by Meder et al.^18^. This methodological choice, simplifies the elicitation method and reduces the amount of trials needed, but could introduce the risk of a description-experience gap^25^. This risk is mitigated (in part) by our methodological choice to have both bets, in a bet couple, carry a degree of risk^26^.

Lastly, by increasing the respondent size (n = 81) but reducing the number of bets (80 choices), we study ensemble effects rather than respondent effects, which offers a different perspective on the findings by Meder et al.^18^.

## Results

### Overall performance in the experiment

At the start of the experiment, we assigned each respondent a random seed. This random seed determined their starting setting (additive or multiplicative), the order in which bet couples and the bets within the bet couples appeared, and whether or not they would receive time pressure. We then informed respondents that they had a starting capital of 1000, which they could increase or decrease by indicating their preferred bet couple for each of the 80 bet couples given in Table 1. After selecting a given bet, one of its two outcomes would randomly (50–50 chances) be added/multiplied with their current capital. Finally, we informed respondents that they would receive no updates on their performance and capital until the end of the experiment. In order to motivate participation and reward engagement, the six respondents with the highest end-capital received one of six cash prizes. To ensure a fair evaluation, all respondents started with the same initial capital (1000), had the same coin flips, and bet order. In the random run employed for distributing the prizes, timed respondents performed slightly better than their counterparts (obtaining a median rank of 34 out of 81). However, because of the high stochasticity of the process and the relatively few trials, this result cannot be generalized.

**Table 1:** All 70 generated bet couples as well as the ‘no-brainer’ bets (index 36–40 and 76–80). Every bet consists of two outcomes with equal likelihood 50/50. Bets within a bet couple would be presented in a random order to each respondent.

Couple (i)	Bet 1 (Riskier)	Bet 2 (safer)	Couple (i)	Bet 1 (Riskier)	Bet 2 (safer)	couple (i)	Bet 1 (Riskier)	Bet 2 (safer)
1	− 308	− 88	28	− 661	461	55	1.26	0.86
	291	71		630	− 492		0.72	1.12
2	− 543	100	29	− 350	− 233	56	1.2	0.79
	554	− 89		334	217		0.71	1.12
3	692	− 254	30	− 331	− 256	57	1.23	0.8
	− 523	423		333	258		0.7	1.13
4	683	− 114	31	340	232	58	0.82	1.19
	− 670	127		− 501	− 393		1.3	0.93
5	− 454	304	32	− 237	− 25	59	1.29	0.93
	481	− 277		303	91		0.88	1.24
6	636	− 371	33	492	45	60	1.29	0.91
	− 549	458		− 454	− 7		0.73	1.11
7	− 394	− 9	34	− 628	− 50	61	1.24	1.14
	367	− 18		456	− 122		0.74	0.84
8	− 232	240	35	− 482	489	62	0.76	0.81
	372	− 100		612	− 359		1.19	1.14
9	524	447	36	190	190	63	1.3	1.22
	− 498	− 421		260	100		0.83	0.91
10	− 183	− 32	37	280	200	64	0.75	0.89
	272	121		− 210	− 210		1.22	1.08
11	446	− 181	38	− 100	20	65	0.78	0.83
	− 344	283		40	− 100		1.2	1.15
12	379	28	39	260	100	66	1.28	0.86
	− 226	125		50	270		0.74	1.16
13	− 226	− 121	40	110	100	67	0.77	0.87
	282	177		50	40		1.28	1.18
14	− 464	229	41	0.71	1.1	68	1.23	0.89
	308	− 385		1.25	0.86		0.73	1.07
15	3	24	42	0.74	0.89	69	1.19	0.85
	121	100		1.23	1.08		0.74	1.08
16	− 673	− 118	43	0.77	0.84	70	1.17	1.09
	629	74		1.18	1.11		0.71	0.79
17	− 235	− 53	44	1.2	1.1	71	1.26	1.14
	74	− 108		0.8	0.9		0.78	0.9
18	− 455	− 147	45	1.23	1.13	72	1.28	1.13
	585	277		0.77	0.87		0.78	0.93
19	− 479	332	46	1.26	0.78	73	0.77	1.15
	523	− 288		0.73	1.21		1.28	0.9
20	542	− 124	47	0.73	1.12	74	0.77	0.84
	− 469	197		1.25	0.86		1.26	1.19
21	56	− 148	48	1.3	0.86	75	1.26	1.16
	− 177	27		0.75	1.19		0.84	0.94
22	66	53	49	1.23	0.95	76	0.71	0.71
	− 190	− 177		0.8	1.08		1.17	1.1
23	− 54	102	50	0.74	0.84	77	0.9	1.3
	128	− 28		1.16	1.06		1.2	0.9
24	− 170	67	51	1.19	0.81	78	0.8	1.1
	237	0		0.73	1.11		1.25	0.7
25	− 82	5	52	1.28	0.92	79	0.72	1.05
	146	59		0.84	1.2		0.95	0.72
26	− 312	143	53	0.77	0.86	80	0.73	1.25
	402	− 53		1.25	1.16		1.25	0.86
27	478	− 39	54	0.83	1.17			
	− 441	76		1.26	0.92			

### The effect of perceived time pressure on response time

**Figure 2:**
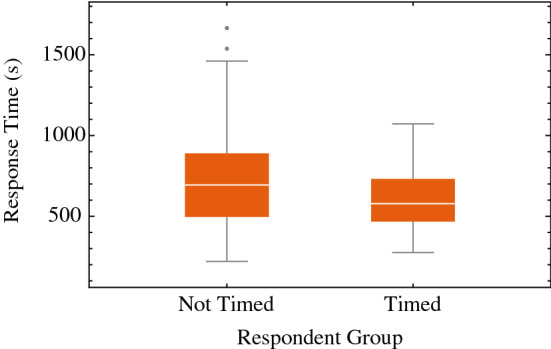
Box plot cumulative bet couple response time (time in seconds). A further decoupling across settings can be found in supplemental, along with the statistical moments.

When testing the effect of perceived time pressure by a non-binding and upward counting clock on response time, we found that the median response time (579 s) for timed respondents was slightly lower than the median response time (694 s) for non-timed respondents (control), as shown in Fig. 2. According to the non-parametric Mann–Whitney test, for our relatively small data set (n = 81), the difference between the two groups is slight (P = 0.042). Additionally, we observed that the group of timed respondents does not vary as much in response time as the non-timed respondent group.

### The effect of perceived time pressure on risk aversion

**Figure 3:**
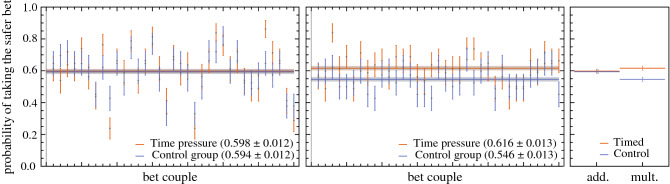
The left panel shows a scatter plot of the estimated probabilities for taking the safer bet for each bet couple in the additive scenario. The overall estimate for this probability for each group, is illustrated by the horizontal error bands. The middle panel is analogous to the left but for the multiplicative scenario. There is a significant difference between the timed and control group in their probability in taking the safer bet, optimizing the average growth rate. The right panel gives a general overview of the difference within both settings.

As described in the methodology section, we estimated the propensity towards the safer bet for all bet couples. Figure 3 depicts the results from this estimation. The *x*-axis indicates the respective bet couple: bet couples 1:35 in the additive and multiplicative setting correspond to bet couples 1:35 and 41:75 in Table 1, respectively. The *y*-axis represents the probability of taking the safer bet. For example, the probability of taking the safer bet for bet couple 1 in the additive setting was $61.3\pm 7.8 \%$ for the timed respondents (orange), and $64.6\pm 7.3 \%$ for the non-timed respondents (blue). We then aggregated the results for each bet couple into an overall probability estimate for both the timed and control groups.

The most exciting difference between the timed and control group lies not in the speed of responses but in their actual answers. In order to contrast the overall response tendency of both groups, we first verified and confirmed the normality of the posterior distribution for all bet couples with the Shapiro–Wilk test. We then estimated the overall probability of taking the safer bet by aggregating the bet couples and propagating their uncertainties. As such, we can then juxtapose the responses of both respondent groups in the additive and multiplicative setting, through the use of a one-sided *z*-test for the aggregated distribution of taking the safer bet. We highlight that we opted for a *z*-test because of the difficulties to compare the concept of risk between the two different settings. We thus set up an experiment with a control group in both settings, which allows us to make a fair comparison within each setting by employing a *z*-test. The control group is the baseline in each setting.

When comparing the posterior distribution of taking the safer bet of the timed respondents versus the non-timed respondents, we noticed that both respondent groups displayed indistinguishable preferences in the additive dynamic. The same could not be said for the multiplicative setting, in which non-timed respondents (control) behaved significantly (P = $7 \times 10^{-5}$) less risk-averse than timed respondents. This is shown in Fig. 3. Classical behavioral economics fails to explain why control and timed respondents behave identically in one dynamic but different in the other. On the other hand, ergodicity economics tells us that both bets were equal in the additive setting, as far as long-time averages were concerned. Consequently, long-time averages had no impact on the decision-making process. However, long-time averages differed in the multiplicative setting, explaining the difference between intuitive decisions and non-intuitive decisions. Despite the possible description-experience gap this is in line with the findings of Meder et al.^18^.

## Discussion

Our results lend support to the theory that intuitive human decision-makers behave differently depending on whether their environment is ergodic or non-ergodic. In agreement with the findings of ergodicity economics, intuitive decision makers tend to optimize the time average of their wealth over the expected values. Despite the differences in methodological choices, our results align with the experiment performed by Meder et al.^18^. More specifically, we do not use images as a proxy for numeric values to induce intuitive choices. Instead, we reinforce intuitive behavior by adding a clock that shows response times. Furthermore, in our paper, we do not need to estimate a utility function to interpret the results. We discuss this point in detail below.

When modeling and interpreting the decision-making displayed by our respondents, we opted to use the risk argument (MC1) rather than extensions on utility theory which takes multiple periods into account. We believe this argument to be appropriate because respondents could not envisage the capital they would have at the end of the experiment and as such could not use this terminal capital as a heuristic within our experiment; they simply had too little information to increase their decision algorithm to multiple periods. Respondents were kept in the dark of the bets that were to come, including the amount of capital they possessed after each decision, bet outcomes, and the order used to determine terminal wealth, which removes the need for dynamic programming^27^. All of this, coupled with an additional cognitive load for 50% of respondents, supports our belief that it is unlikely that decision makers considered more than just the information right in front of them.

When evaluating control versus timed respondents, we observed that while response time was not greatly impacted (P = 0.042), voting behavior was. As such, we believe that time pressure had its intended effect. Namely, it increased the cognitive load on respondents, making their answers more intuitive^28,29^ than those of the control respondents. Our findings regarding time pressure hint at a relation between the impact of time pressure on decision making, the bet dynamic, and setting. Timed respondents display a significantly (P=$7 \times 10^{-5}$) greater preference for safe bets than their non-timed counterparts in the general multiplicative environment, which partly supports the findings of Zur et al.^24^. In contrast, time pressure seemed to have little or no impact on risk attitudes within the general additive setting. This surprising result implies that intuitive respondents (timed) came closer to employing the optimal strategy than the respondents in the control group. This is in line with predictions from ergodicity economics which states that people naturally tend towards optimizing the time average rather than the expected value. This provides additional evidence that human decision-makers use time-optimizing models in some circumstances. Nevertheless, the range of human decision-making can be different in many different circumstances. When appropriate, it can use other models (time or ensemble averages) such as, e.g., Kool et al.^30^ and McDermott et al.^31^.

Further experimentation is required to solidify this statement, as it could be the result of the formal training our respondents received regarding expected values. We will address this limitation in future research on a more diverse group of participants. However, the formal training of our respondents can not explain the difference in reliance on expected values within different dynamical settings. Some additional avenues for further research would be an experiment with bet couples in which either purely positive or negative outcomes, or an experiment in which the odds from each outcome are varied while keeping the expected values the same. These experiments would allow for a closer inspection of ergodicity economics versus the most replicable results from prospect theory^32^. The same experiment could also be reproduced but with different starting capitals to ascertain the consequences of possible wealth effects.

Based on our findings, we conclude that intuitive decision makers tend to base their decisions to a greater extent on long-time averages than expected values. This link between long-time averages and intuition supports the idea that human decision-making is based on a non-ergodic process. This is not to argue that human decision-making is perfectly rational, but rather that as proposed by ergodicity economics, a different variable—time averages, in particular—should be considered when modeling human decision makers. It is interesting to note that to act in a time-optimal manner is different from estimating exponential processes. Some evidence argues that humans are bad at doing that^33,34^. However, to act time optimally, one does not need to estimate the global process, it suffices to think locally (in time). To sum, our findings highlight that ergodicity economics offers an essential insights into the interpretation of human decision-making, and that we should be critical of the currently employed definitions of rationality. Where rationality is often defined with respect to subjective utility functions.

## Methods

To increase sample size, we opted for an online experiment rather than a physical experiment. In this experiment, each respondent was assigned €1000 as starting capital. Throughout the experiment, each respondent was given a choice to invest their capital into one of two bets and this 80 times over, while being subjected to two different dynamics. In the additive dynamic, bet outcomes would be added to their capital. In the multiplicative dynamic, outcomes would be multiplied with their capital. First, we will discuss the method used to distinguish between risk-averse and risk-taking decisions. In the second part, we build on this to algorithmically generate the bets used throughout the experiment. In the third part, we describe the sampling procedure, and fourthly we describe our data analysis.

### Methodological choices: (1) Fit the behaviour, not the entire utility function

Rather than opting for a specific utility function, we decided to focus on how often a respondent behaves risk-averse. Therefore, we designed bet couples with the following characteristics: each bet in a given couple had the same expected outcome but a different variance. Consequently, opting for the couple with higher variance indicates a risk-taking decision, and opting for the lower variance signifies a risk-averse decision.

We can strengthen the aforementioned claim if we assume that the utility function for each respondent has a constant second order derivative, and is monotone—i.e., gaining more or losing less is always better than gaining less or losing more. These assumptions coupled with Jensen’s inequality^35^ allows us to classify any two bets according to their curvature and the insights from utility theory^13^: concave utility functions describe risk averse decision makers, convex utility functions describe risk taking decision makers, and linear utility functions describe risk neutral decision makers. As such, we reduced the question of risk averseness to a binary one for any monotone utility function, the stated preference implies either a concave or convex utility function. For example, consider two bet couples, Bet$_1$ and Bet$_2$. Bet$_1$ has a probability of *p* for its least favourable outcome *a*, and probability $1-p$ for its most favourable outcome *d*. Bet$_2$ has the same expected outcome as Bet$_1$ but with more moderate outcomes *b* and *c* with a probability of *q* and $1-q$ respectively. If a respondent prefers Bet$_2$ over Bet$_1$ this implies a concave utility function and as such risk averse behaviour. This can be shown using standard mathematical techniques, the proof of which is provided below. A fortunate consequence of using bets with equal expected values is that it allowed us to distinguish the bet with the highest time average with relative ease. In the additive scenario, because of ergodicity, both bets had an equal long-time average. In the multiplicative setting, the bet with the lowest variance had the highest time average.

#### Lemma 0.1

*Given 2 bets Bet*$_{1}=[(p,a);(1-p,d)]$ *and Bet*$_{2}=[(q,b);(1-q,c)]$ *with equal expected outcomes, i.e.* $p a+(1-p)d=q b+(1-q)c$ **(1)**, *for which* $a<b<c<d$. *If Bet*$_2$ *is preferred over Bet*$_1$ (Bet$_{2} \succeq$Bet$_{1}$), *the utility function* *U*(*x*) *is monotone* $\forall x\in \left[ a,d\right]$, *and* $sgn(U''(x))$ *is constant* $\forall x\in \left[ a,d\right]$, *it follows that* *U*(*x*) *is concave* $\forall x\in \left[ a,d\right]$.

#### Proof

Given Bet$_{2} \succeq$Bet$_{1}$. So,

$$\begin{aligned}&pU(b)+(1-p)U(c) \ge qU(a)+(1-q)U(d) \\&\implies pU(b)+(1-p)U(c)-U( b+(1-p)c) \ge qU(a)+(1-q)U(d)-U( b+(1-p)c) \\&\implies pU(b)+(1-p)U(c)-U( b+(1-p)c) \ge qU(a)+(1-q)U(d)-U( qa+(1-q)d)&\mathbf (1) \\&\implies U(x) \hbox {is concave} \forall x\in \left[ a,d\right] \qquad \qquad (\text {Jensen's Gap argument}) \\ \end{aligned}$$

$$\square$$

### Methodological choices: (2) Defining the bet parameter space

**Figure 4:**
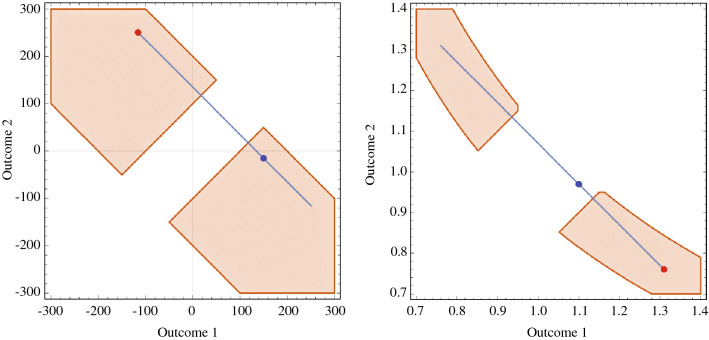
The parameter space for the bet couples, with the additive scenario shown on the left and the multiplicative scenario shown right. A bet couple is generated as follows. First, our algorithm selects a random point in the orange area. In this example, the algorithm picked the red dot. Second, the algorithm selects a random point anywhere on the blue line (this includes the white region between the orange areas), which represents all the bets with equal expected value as the red dot but less variance. In this example, the algorithm picked the blue dot. Together, the red and blue dot make up one bet couple.

The choices described in the previous subsection allowed us to algorithmically create bets for both the additive and the multiplicative settings. To ensure that the generated bets were not too similar and would not make capital explode or plunder to zero, we restricted the parameter space as follows (see Fig. 4): In the additive setting, bet outcomes could not result in a loss or gain greater than 300. The expected bet gains or losses were equally restricted in that they could not be greater than 100. In the multiplicative setting, bet outcomes could not be greater than 1.4 or smaller than 0.7, and the long-time average growth rates for each bet could not be greater than 1.05 or less than 0.945. The algorithm could then freely select any point in the orange areas in Fig. 4, resulting in a first bet. Next, we constructed the blue line, representing all bets with equal expected value but different variance. The algorithm then selected any point on the blue line, resulting in a bet couple. All 70 generated bet couples can be found in Table 1. As a sanity check, we created five extra ‘no-brainer’ bet couples for each setting. Respondents needed to correctly identify at least 3 out of 5 ‘no-brainer’ bets for each setting to be considered for analysis. Of the initial 100 respondents, 81 met this criterion, and 78% of all answered ‘no-brainer’ bets were correctly identified.

### Methodological choices: (3) Respondents, implementation, and rewards

Respondents were invited based on a convenience sample from a relatively homogeneous population. All targeted respondents were first- and second-year Economics students from the same university (Vrije Universiteit Brussel). All of which enjoyed (at least) introductory classes to mathematics, statistics, micro-, and macroeconomics. All of the experimental procedures were conducted in compliance with the ethical regulations of the Vrije Universiteit Brussel. All experimental protocols were approved by the Ethics Committee for Human Sciences of the Vrije Universiteit Brussel (ECHW). All subjects were legal adults who gave their informed consent to participate in the study. Participants provided their e-mail address and completed the task online. Besides the outcome of the task and e-mail address, we collected no other information. After the completion of the experiment, we deleted all of the e-mail addresses.

Analogous to Meder et al.^18^, the survey was split into two independently played parts, i.e., a respondent assigned to the additive setting would first have to terminate this part of the experiment before being able to move on to the next part and visa versa. At the start of the survey, each respondent was told that they had a starting capital of €1000 and that they would receive no further updates about their capital and the obtained bet outcomes. Subsequently, each respondent was randomly assigned a starting setting, bet, and one of the two groups: with or without time pressure. To ensure that respondents correctly understood the different settings, a written explanation, an example, and an instruction video were provided before each part of the experiment. Time pressure was introduced in the form of a timer next to the given bet couple, which started counting up as soon as a new bet couple appeared. This clock merely served as a psychological tool to encourage intuitive decision making and, as such, had no consequences tied to it^28,29^, which means that all time pressure experienced from this clock was only perceived.

In order to motivate respondents and mitigate the excess risk-taking linked to winner-take-all games, six prizes could be won^36^. These prizes were given to the respondents who obtained the highest capital at the end of the experiment. All respondents started with the same amount of money, had the same coin flips, and the same standardized bet order when calculating end capital.

### Methodological choices: (4) Averaging over people

Following the curvature argument, decisions were categorized as either risk-taking (coded 0) or risk-averse (coded 1). Therefore, we could describe the underlying process of choosing the safer bet over the riskier bet using the binomial distribution, for which $p_{i}$. (probability of taking the safe bet in bet couple) needed to be estimated.

$$\begin{aligned} \left( {\begin{array}{c}n\\ k\end{array}}\right) p_i^k\left( 1-p_i\right) ^{n-k}\ \end{aligned}$$

As a method for estimating $p_{i}$, we used naive Bayesian updating. For this, we set up a two-step process to find the probability distribution of $p_{i}$. At the outset of each updating process, no prior knowledge was assumed. We started by estimating an appropriate prior probability distribution by using 80% of the observations for bet couple *i*, in tandem with Jeffrey’s prior (least informative prior)^37^. This initial update resulted in a more informed prior probability distribution. We then used the obtained distribution as a prior for the Bayesian updating process to which the remaining 20% of observations were iteratively added, engendering our estimate probability distribution of $p_{i}$ which are approximately normally distributed (according to both Shapiro–Wilk and Jarque–Bera test). As a reminder, our studied setting is the difference between timed and non-timed respondents for both the additive and multiplicative setting. For the these two scenarios of the given setting, we approximated the setting-dependent $p_{s}$ by averaging over the $p_{i}$ related to that particular setting.

## Supplementary Information

Supplementary Figure 1.

## Data Availability

The computer code and data have been made available at: https://github.com/Arne-Vanhoyweghen/Ergodicity-Stated-Choice.
