# Supplementary material for: The influence of ergodicity on risk affinity of timed and non-timed respondents
Source: Sci Rep. 2022 Mar 8;12:3744. doi: 10.1038/s41598-022-07613-6 (PMC8904497; doi:10.1038/s41598-022-07613-6)
Supplement: Supplementary file 1 — Supplementary Figure 1. [file 41598_2022_7613_MOESM1_ESM.pdf]

Supplemental Material to: The influence of ergodicity on risk affinity of timed and non-timed respondents

Arne Vanhoyweghen[1,2,*], Brecht Verbeken[2], Cathy Macharis[3], and Vincent Ginis[1,4]

[1]Data Lab, Vrije Universiteit Brussel, Brussels, 1050, Belgium
[2]Business technology and Operations, Vrije Universiteit Brussel, Brussels, 1050, Belgium
[3]Mobility, Logistics, and Automotive Technology Research Centre, Vrije Universiteit Brussel, Brussels, 1050, Belgium
[4]School of Engineering and Applied Sciences, Harvard University, Massachusetts, 02138, USA
[*]arne.vanhoyweghen@vub.be

Supplemental figure

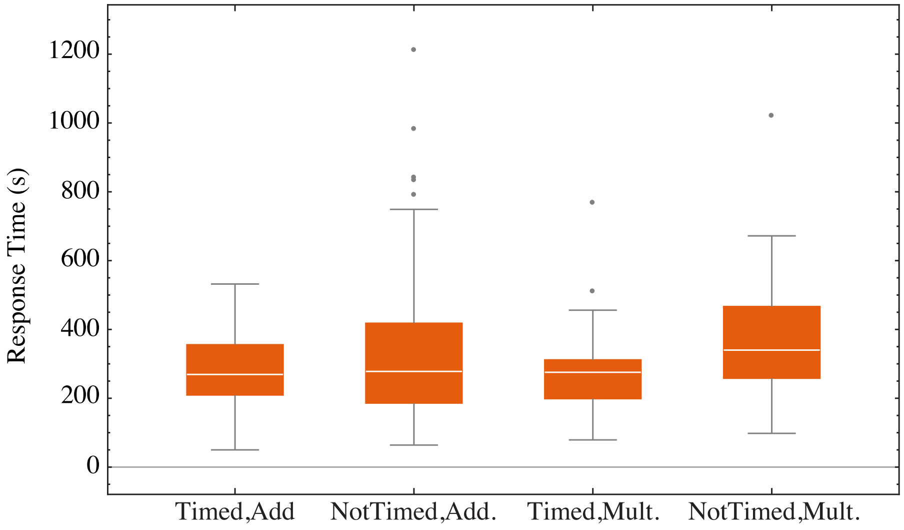

Supp. Figure 1. Box plot cumulative bet couple response time (time in seconds) for each of the uncoupled settings. The statistical moments for each respective setting are as follows: Timed Additive ($M_1 : 280, M_2 : 11865, M_3 : 0.41,$ and $M_4 : 3$), Not Timed Additive ($M_1 : 362, M_2 : 70000, M_3 : 1.48,$ and $M_4 : 4.5$) , Timed Multiplicative ($M_1 : 284, M_2 : 15952, M_3 : 1.46,$ and $M_4 : 6.8$), and Not Timed Multiplicative ($M_1 : 370, M_2 : 33053, M_3 : 1.19,$ and $M_4 : 5.3$). A simple one way ANOVA test yields a p-value of 0.035, which does not provide strong evidence for equal means.
